# Postoperative Home Monitoring After Joint Replacement: Feasibility Study

**DOI:** 10.2196/10168

**Published:** 2018-09-05

**Authors:** Homer Yang, Geoff Dervin, Susan Madden, Paul E Beaulé, Sylvain Gagné, Mary L Crossan, Ashraf Fayad, Kathryn Wheeler, Melody Afagh, Tinghua Zhang, Monica Taljaard

**Affiliations:** 1 Department of Anesthesia & Perioperative Medicine Schulich School of Medicine Western University London, ON Canada; 2 Division of Orthopaedic Surgery University of Ottawa Ottawa, ON Canada; 3 Department of Nursing The Ottawa Hospital Ottawa, ON Canada; 4 Department of Anesthesia and Pain Medicine University of Ottawa Ottawa, ON Canada; 5 Department of Anesthesia and Pain Management The Ottawa Hospital Ottawa, ON Canada; 6 Clinical Epidemiology Program Ottawa Hospital Research Institute Ottawa, ON Canada; 7 School of Epidemiology and Public Health University of Ottawa Ottawa, ON Canada

**Keywords:** postoperative home monitoring, postoperative transitional care, surgical length of stay, postoperative wireless monitoring, patient confidentiality during wireless monitoring, mobile phone

## Abstract

**Background:**

We conducted a prospective observational study of patients undergoing elective primary hip or knee replacements to examine the feasibility of a postoperative home monitoring system as transitional care to support patients following their surgery in real time.

**Objective:**

The primary outcome was the mean percentage of successful wireless transmissions from home of blood pressure levels, heart rate, oxygen saturation levels, and pain scores until postoperative day 4 with a feasibility target of ≥90%.

**Methods:**

Patients with an expected length of stay ≤1 day, age 18-80 years, Revised Cardiac Risk Index ≤ class 2, and caretakers willing to assist at home were eligible. Patient satisfaction, as a secondary outcome, was also evaluated. Wireless monitoring equipment (remote patient monitoring, Telus Canada) was obtained and a multidisciplinary care team was formed.

**Results:**

We conducted the study after obtaining Research Ethics Board approval; 54 patients completed the study: 21 males, 33 females. In total, we evaluated 9 hips, 4 hip resurfacing, 26 total knees, and 15 hemi-knees. The mean transmission rate was 96.4% (SD 5.9%; 95% CI 94.8-98.0). The median response to “I would recommend the Remote Monitoring System program to future patients” was 4.5 (interquartile range 4-5), with 1 being “strongly disagree” and 5 “strongly agree.” At 30 days postop, there was no mortality or readmission.

**Conclusions:**

This is an evolving new paradigm for postoperative care and the first feasibility study on monitoring biometrics after primary hip or knee replacement. Postoperative home monitoring combines current technology with real-time support by a multidisciplinary transitional care team after discharge, facilitating postsurgical care with successful wireless transmission of vitals. The postoperative home monitoring implementation is, therefore, generalizable to other surgical discharges from hospitals.

**Trial Registration:**

ClinicalTrials.gov NCT02143232; https://clinicaltrials.gov/ct2/show/NCT02143232 (Archived by WebCite at http://www.webcitation.org/71ugAhhIk)

## Introduction

### Background

For a number of reasons, including the impetus to increase surgical throughput, the median length of stay in Canada has been decreased from 6 days in 2006-2007 to 4 days in 2012-2013 for total hip replacements and from 5 days in 2006-2007 to 3 days in 2013-2014 for total knee replacements [1,2]. Nevertheless, the Canadian Institute of Health Information data (on hip or knee arthroplasty) suggest that “demand is rising at a rate that is outpacing the ability of health systems to keep up” [3].

The literature shows that although most patients have no surgical “returns” such as emergency department (ED) visits or readmissions within 30 days of surgical discharge, 6.5% are readmitted and 18.7% return to the ED within this period in Canada [4]. In one study in the United States, the 30-day readmission rate after total knee replacement was reported to be 5.6% [5]. In another study, the 30-day complication rate after hip or knee replacement was reported to be 2%, with complications including myocardial infarctions, deep vein thrombosis, pulmonary embolism, and death [6]. It is important to note the corollary that 98% of patients did not have complications and that 95.4% were not readmitted within 30 days of hip or knee replacements. The statistics, therefore, support the concept of earlier discharge in spite of a small proportion of patients requiring readmission.

Data from the Canadian Institute of Health Information also show that 1.9%, 9.4%, and 18.7% of postsurgical patients visited the ED within 1, 7, and 30 days of discharge, respectively (based on Ontario, Alberta, and Yukon data) [4]. Of the postsurgical patients who visited the ED within 7 days of discharge, 28.3% (8363/29,552) were evaluated to be at Canadian Triage and Acuity Scale (CTAS) level IV or V, that is, nonlife-threatening or emergent conditions. Such ED visits were potentially preventable or manageable at home.[4] In contrast, 24.4% and 47.2% of postsurgical visits to the ED were emergent and urgent (CTAS I, II, and III), respectively. Delay in taking such patients to the original index hospital results in increased mortality and costs [7,8]. The challenge is, thus, to decide which patients need to be repatriated expeditiously after discharge versus the ones with lesser complications to be managed at home. The postoperative home monitoring (POHM) solution allows remote wireless transmission of blood pressure (BP) levels, heart rate (HR), oxygen saturation (SpO_2_) levels, and pain scores using a tablet, noninvasive blood pressure cuff, and Bluetooth saturation monitor. We hypothesize that using this monitoring system, patients could be wirelessly monitored at home and their concerns after discharge may be addressed to appropriately. This is a report of an outpatient hip and knee replacement pathway at our institution using POHM.

### Objectives

The objectives of this study were to demonstrate the feasibility of wireless home monitoring after elective primary hip or knee replacements with a primary feasibility target of ≥90% successful transmission of BP levels, HR, and SpO_2_ levels and to collect pain scores 4 times a day from home until postoperative day (POD) 4. Secondary outcome included patient satisfaction.

## Methods

Approval from the Ottawa Hospital Research Ethics Board was obtained for a prospective observational study (NCT02143232) of patients undergoing elective primary hip or knee replacements with an expected length of stay ≤1 day (same day discharge), age between 50 and 80 years, Revised Cardiac Risk Index ≤Class 2, and caretakers to assist at home. As the study progressed, a younger age group was found to present for primary hip or knee replacements, which prompted a change in our age inclusion criterion from 50-80 years to 18-80 years, and we obtained an additional Research Ethics Board supplemental approval. Exclusion criteria included the presence of American Society of Anesthesiology Class IV, chronic obstructive pulmonary disease with forced expiratory volume 1 second ≤1, obstructive sleep apnea, patient or family reluctance to participate in early discharge, prior enrollment in POHM, and a disease process that was unstable or undiagnosed. A sample size of 54 was sufficient to yield a one-sided 95% CI estimate around our primary outcome measure (proportion of successful transmissions) with a lower bound exceeding the cut-off point for feasibility of 90%, assuming a proportion of 95% successful transmissions. Consent was obtained in the Preadmission Unit (PAU) starting in March 2014 as per the Ottawa Hospital Research Institute standard operating procedures. The choice of anesthetic was left at the discretion of the anesthesiologist assigned to the case. Surgical approach was as per standard practice of minimally invasive technique: direct anterior in the hip or subvastus in the knee. Patients followed the standard postanesthetic recovery unit’s hip and knee replacement clinical pathways.

Prior to discharge on the same day of surgery, remote patient monitoring (RPM, Telus Canada) hardware with cellular connectivity to the patient’s home, alerts to the research team’s smartphones, and data storage behind the hospital firewall were set up. A care path for primary hip or knee replacement was defined, with acetaminophen, celecoxib, an opioid (tapentadol, tramadol, or hydromorphone), pregabalin, and an anticoagulant (apixaban or rivaroxaban) prescribed on discharge unless otherwise contraindicated. Monitoring of BP, HR, SpO_2_, and pain scores was performed 4 times a day for 4 days postoperatively, and the data obtained were transmitted to the hospital server behind the firewall. Specific alert protocols were set up within the software (Telus, Canada), and a primary responder from within the research team was designated to receive the alerts at all times. Otherwise, the primary responder would check the Web-based monitoring dashboard once a day.

A patient questionnaire using a 5-point Likert scale (1: “strongly disagree,” 3: “neutral,” and 5: “strongly agree”) was administered using the hardware (RPM, Telus Canada) without any research personnel present at the end of each monitoring period. Patients were followed up on POD 5 and via phone call on POD 30. Descriptive statistics (mean and SD or frequency and percentage) were used to describe the preoperative and predischarge characteristics of participants. Mean, SD, and median transmission rates were used to describe the actual transmissions over the total daily possible transmissions. Mean and SD were used to describe responses to the patient questionnaire. We followed the Strengthening the Reporting of Observational Studies in Epidemiology statement in reporting this study.

## Results

**Figure 1:**
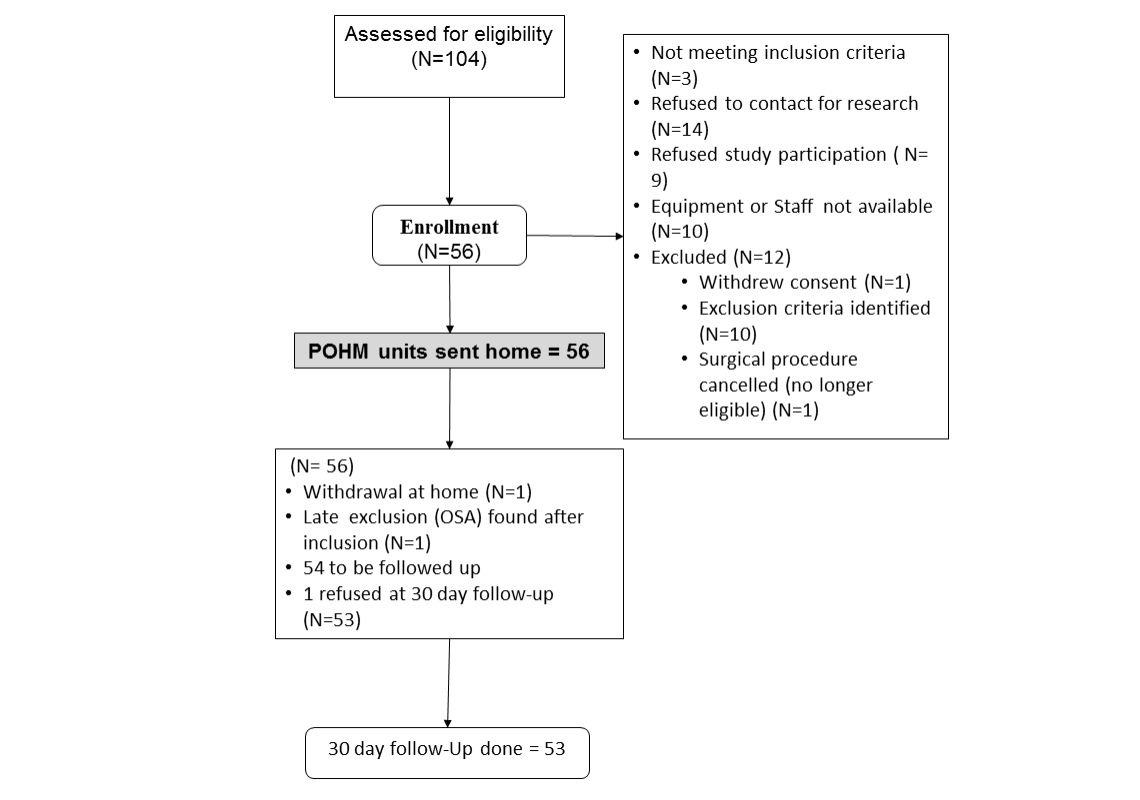
Recruitment diagram for postoperative home monitoring (POHM) part 1. OSA: obstructive sleep apnea.

The target sample size of 54 patients was achieved between April 2014 and September 2015. Patients’ demographic characteristics and comorbidities are presented in Table 1. Patients’ eligibility, recruitment, and participation in the study are shown in the flowchart (Figure 1). Surgical procedures, anesthetic type, and medications received are reported in Table 2. The overall mean transmission rate was 96.4% (SD 5.9%; 95% CI 94.8-98.0), and the median transmission rate was 97.9% (interquartile range [IQR] 97.8%-98.8%; Table 3). There were 6 alerts of BP>140 mm Hg, 7 of BP<90 mm Hg; 7 alerts of HR>120 beats per minute, 0 of HR<50 beats per minute; and 1 alert of SpO_2_ 88% (ie, SpO_2_<90%). “Unsatisfied with pain control” alerts were sent by patients on 7 occasions and “pain limiting movement” alerts on 13 occasions. Apart from the courtesy phone call made on the evening of discharge, the median number of phone calls to patients during the 4 days of monitoring was 1.0 (IQR 1-3), with 11 and 21 patients with 0 or 1 phone call, respectively; 8 patients required 5 phone calls during the 4 days of monitoring. There was no mortality in the 30-day postoperative period.

**Figure 2:**
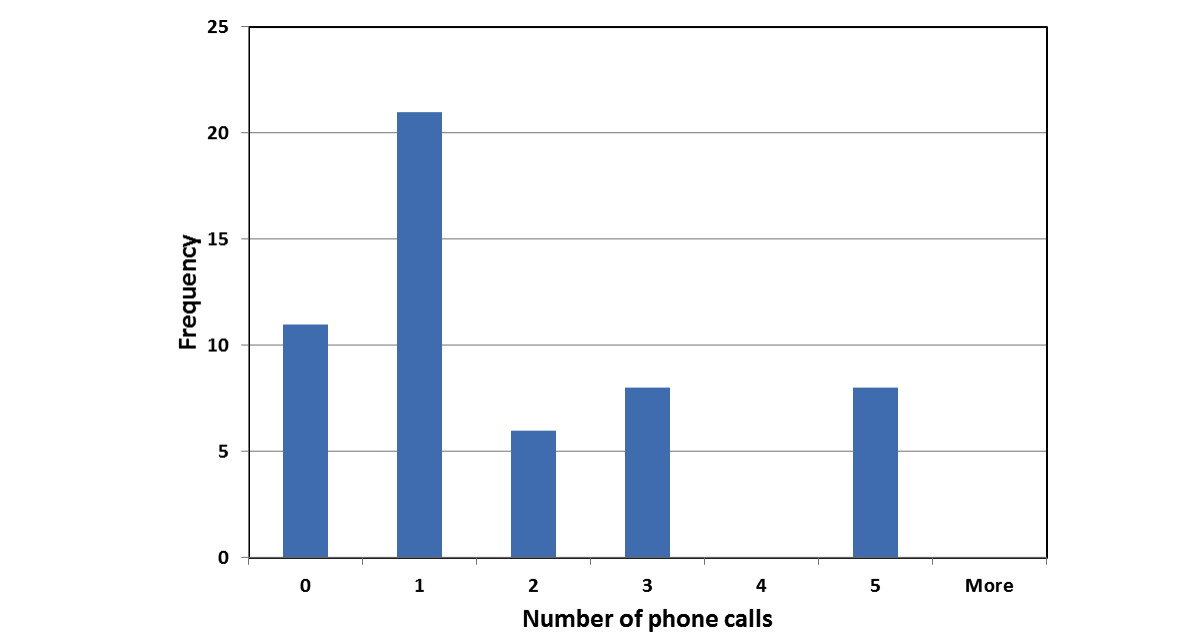
Frequency of phone calls during the 4 days of monitoring, part 1.

Table 4 shows the patient responses to the questionnaire at the completion of the home monitoring. The median response to “I would recommend the Remote Monitoring System program to future patients” was 4.5 (IQR 4-5), with 5 being “strongly agree” (Figure 2). At the end of the monitoring questionnaire, patients were provided the opportunity to provide further comments (Table 5).

**Table 1:** Patient demographics.

Variable		Patients (N=54)
Age (years), mean (SD)		61.4 (8.3)
**Sex, n (%)**		
	Female	33 (61)
	Male	21 (39)
Body mass index (kg/m^2^), mean (SD)		27.51 (4.0)
**American Society of Anesthesiology Class, n (%)**		
	I	5 (9)
	II	40 (74)
	III	9 (17)
	IV	0 (0)
High blood pressure on treatment, n (%)		15 (28)
Type II diabetes mellitus on treatment, n (%)		3 (6)
Hypercholesterolemia on treatment, n (%)		14 (26)
Preoperative nonsteroidal anti-inflammatory drug use, n (%)		23 (43)
Current smoker, n (%)		3 (6)

**Table 2:** Surgical procedures, anesthetic type, and medications received.

Variable		Patients (N=54), n (%)
**Surgical procedure**		
	Total hip	9 (17)
	Hip resurfacing	4 (7)
	Total knee	26 (48)
	Hemi knee	15 (28)
**Anesthesia type**		
	Spinal	50 (93)
	General anesthesia	4 (7)
**Medication received**		
	Nonsteroidal anti-inflammatory drug on discharge	40 (74)
	Tapentadol or tramadol on discharge	14 (26)
	Acetaminophen on discharge	49 (91)
	Pregabalin on discharge	54 (100)
	Opioid on discharge	38 (72)
	Anticoagulant on discharge	51 (94)

**Table 3:** Transmission rates during the first 4 days postoperatively.

Transmission	Percent mean (SD)
Transmission on day of Sx^a^	99.5 (0.03)
Transmission on postoperative day 1	98.3 (0.06)
Transmissions on postoperative day 2	97.9 (0.06)
Transmissions on postoperative day 3	97.8 (0.06)
Transmissions on postoperative day 4	90.9 (0.24)
Transmission per day overall^b^	96.4 (5.9)

^a^Day of Sx: Four transmissions (blood pressure [BP], heart rate [HR], oxygen saturation [SpO_2_], and pain); postoperative days 1-4: (BP, HR, SpO_2_, pain) × 4 per day × 4 days; total possible transmissions: 68 per patient during the study.

^b^95% CI 94.8-98.0.

**Table 4:** Patient satisfaction survey (postoperative day 5).

Variable	Mean (SD)^a^	Number of patients answering the question^b^
*“The information provided, told me what to expect about the Remote Monitoring System at home.”*	4.57 (0.54)	51
*“The instructions on how to set up and use the Remote Monitoring System were easy to understand.”*	4.61 (0.57)	51
*“The Remote Monitoring System was difficult to use.”*	1.82 (0.87)	51
*“I felt safe at home during the four days of monitoring.”*	4.33 (1.01)	51
*“During the 4 day monitoring, the response by the Clinician was efficient.”*	4.46 (0.89)	50
*“There was too much to manage at home including the Remote Monitoring System.”*	2.22 (1.19)	51
*“The length of four days for the actual monitoring was just right.”*	4.14 (0.72)	51
*“During the 4 day monitoring, I would have liked more feedback from the Clinician.”*	2.41 (1.1)	51
*“I would recommend the Remote Monitoring System program to future patients.”*	4.36 (0.8)	50

^a^1: “strongly disagree,” 5: “strongly agree.”

^b^Not every patient answered every question.

**Table 5:** Patient comments.

Patient	Comment
#1	*“Excellent wonderful.”*
#2	*“Excellent, godsend.”*
#10	*“Comforting-monitoring remotely triggered interaction when at home as he had event. He had event, low BP. He would like comment section at each evaluation time to express how one feels.”*
#26	*“Very good - reassuring that clinician sees the results entered BP monitor should have been demonstrated more with husband.”*
#40	*“very good. Pain episode was managed,,hard to remember the time but by Day 2 ok, Preop stressed and at discharge but having wife shown equip was good.”*
#44	*“concerned how pills affect you .wonderful, very safe, good to check blood pressure.”*

## Discussion

Our results demonstrate the feasibility of POHM at a transmission rate of >96% supported by a response team. Early data transmission and clear communications between the patients and response team led to alteration in the postoperative course. This is clearly demonstrated from a patient’s comment:

“i had a pain crisis on day 2 and this programme allowed me to speak directly with [the nurse] and receive instructions and her rechecking on me I am immensely grateful to her and her initiative. [M]y only other re course would have been a trip to emergency and wait in line. This programme provides an indispensable safety net for major surgery day patient well done.”

In an analysis mainly on chronic disease management in a pan-Canadian study on RPM in 2014, acute care was considered and thought to be the most complex of the RPM initiatives, at level 5 [9]. In the risk stratification framework, RPM deployment should ensure that technological complexity, patient acuity, and risk of hospitalization (rehospitalization in our case) are aligned. A patient profile with moderate to high risk of (re)hospitalization should be known to one or more services to ensure multidisciplinary case management. We concur with the conclusion and having a multidisciplinary team; our care model involved surgery, anesthesia, acute pain service, and nursing.

The importance of the POHM is that it does not rely only on the availability of software and hardware but also on the infrastructure to support the home monitoring, including patient safety, secure transmissions, and team response while maintaining privacy. Potential data security and privacy breaches are an increasing concern in mobile medicine [10,11]. One study identified potential data security and privacy breaches in 95.63% (17,193/17,979) of mobile iOS apps [12]. In our project, patient confidentiality and data security were built into the design from the beginning, starting with the hospital firewall for data repository and the use of protected institutional emails. We believe it to be of paramount importance, and since the study completion, we have continued the project in partnership with the Ontario Telehealth Network, which has data infrastructure in compliance with the provincial Office of the Information and Privacy Commissioner of Ontario.

In addition, a primary responder should be designated at the originating hospital to review patients’ surgical and anesthetic histories whenever alerts are received. The protocols for alerts include algorithms to allow an escalation of severity. The immediate transmission of alerts to the primary responder’s smartphone allows the primary responder not to be tied to a monitor but be able to carry out other duties during the monitoring period. In addition, as demonstrated in our feasibility study, most of the patients, in fact, only required 0 or 1 phone call over the 4 days apart from the initial courtesy call on the day of discharge. Nevertheless, 8 of 54 patients required 5 phone calls over the 4 days for support and management. The escalating alert algorithm allows the primary responder to focus on the patients who require more attention at home after discharge.

There have been studies on postsurgical RPM; however, all but one study were on the monitoring of activity levels at home using mobile devices such as smartphones [13-15]. The one study in which bio signs were monitored at home was on 20 patients who had undergone liver transplantation [16]. We present here the first feasibility results on POHM of bio signs after primary hip or knee replacements.

There are some limitations to the current study. It was a prospective, observational trial without interventions. The primary outcome was collected using actual digital transmissions to the hospital server as an objective count. The patient questionnaire was administered at the end of the monitoring period using POHM hardware at the patients’ home without any researcher being present. It is unlikely that a bias would have influenced patients’ responses. The actual data on 30-day mortality and any other adverse events were collected by the research team via phone calls, and being a numerical count, the data were objective and unbiased. We believe, therefore, that the feasibility and reliability of POHM were demonstrated without bias.

Any surgical population with low surgical readmission or ED visit rates would be an excellent candidate for earlier discharge and POHM. In other surgical specialties, initiatives such as Early Recovery after Surgery have been implemented to achieve earlier discharge [17]. With the advent of minimally invasive surgery, improved anesthetic techniques, and postoperative pain management modalities, earlier postsurgical discharge is increasingly possible and appropriate; POHM is, therefore, generalizable to other surgical populations.

Our study demonstrated that a wireless system is feasible for monitoring patients at home postoperatively. Combining real-time interactive support by the health care team and the rapidly evolving monitoring technologies such as wearables, POHM systems hold great promise for even more advanced monitoring at home. The automated system with escalating alerts is a monitoring system with built-in intelligence and allows the primary responder to monitor patients without being tied to a monitor. We believe that POHM is a new paradigm of transitional care for surgical recovery in the postacute care period.

## Abbreviations

BP: blood pressure
ED: emergency department
HR: heart rate
POD: postoperative day
POHM: postoperative home monitoring
RPM: remote patient monitoring
